# Diagnostic reasoning in neurogenetics: a general approach

**DOI:** 10.1055/s-0042-1755275

**Published:** 2022-11-09

**Authors:** Helena Fussiger, José Luiz Pedroso, Jonas Alex Morales Saute

**Affiliations:** 1 Universidade Federal do Rio Grande do Sul, Faculdade de Medicina, Programa de Pós-Graduação em Medicina: Ciências Médicas, Porto Alegre RS, Brazil.; 2 Universidade Federal de São Paulo, Departamento de Neurologia, Unidade de Ataxias, São Paulo SP, Brazil.; 3 Hospital de Clínicas de Porto Alegre, Centro de Pesquisa Clínica, Neurogenética, Porto Alegre RS, Brazil.; 4 Hospital de Clínicas de Porto Alegre, Serviço de Genética Médica, Porto Alegre RS, Brazil.; 5 Hospital de Clínicas de Porto Alegre, Serviço de Neurologia, Porto Alegre RS, Brazil.; 6 Universidade Federal do Rio Grande do Sul, Faculdade de Medicina, Departamento de Medicina Interna, Porto Alegre RS, Brazil.

**Keywords:** Neurology, Genetics, Medical, Diagnosis, Neurologia, Genética Médica, Diagnóstico

## Abstract

Establishing the definitive diagnosis of a neurogenetic disease is usually a complex task. However, like any type of clinical diagnostic reasoning, an organized process of development and consideration of diagnostic hypotheses may guide neurologists and medical geneticists to solve this difficult task. The aim of the present review is to propose a general method for diagnostic reasoning in neurogenetics, with the definition of the main neurological syndrome and its associated topographical diagnosis, followed by the identification of major and secondary neurological syndromes, extraneurological findings, and inheritance pattern. We also discuss general rules and knowledge requirements of the ordering physician to request genetic testing and information on how to interpret genetic variants in a genetic report. By guiding the requests for genetic testing according to an organized model of diagnostic reasoning and with the availability of specific treatments, clinicians may find greater resoluteness and efficacy in the diagnostic investigation, shortening the struggle of patients for a definitive diagnosis.

## Resumo

Estabelecer o diagnóstico definitivo de uma condição neurogenética geralmente é uma tarefa complexa; entretanto, semelhante a qualquer raciocínio diagnóstico clínico, um processo organizado de formulação e ponderação de hipóteses diagnósticas pode ajudar neurologistas e médicos geneticistas a resolverem essa difícil tarefa. O objetivo desta revisão é propor um método geral de raciocínio diagnóstico em neurogenética, com a definição da síndrome neurológica principal e seu diagnóstico topográfico associado, seguidos da identificação das síndromes neurológicas principais e secundárias, dos achados extraneurológicos, e do padrão de herança. Também discutimos as regras gerais e os requisitos de conhecimento do médico solicitante para o pedido de teste genético e informações sobre como interpretar variantes genéticas quando recebemos um laudo. Ao orientar a solicitação de exames genéticos de acordo com um modelo organizado de raciocínio diagnóstico e com a disponibilidade de tratamentos específicos, o clínico poderá encontrar maior resolutividade e eficiência na investigação diagnóstica, o que encurtará a odisseia do paciente para um diagnóstico definitivo.

## INTRODUCTION

**Figure 1:**
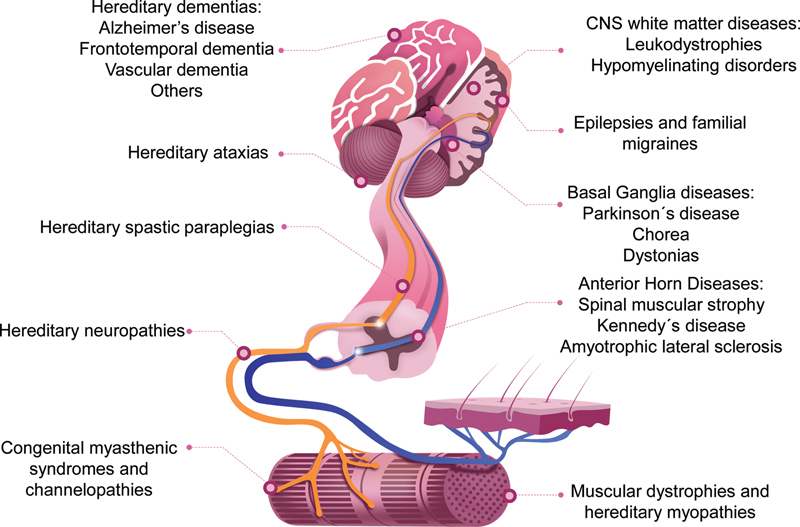
Major groups of neurogenetic conditions according to the topographical diagnosis.

Clinical neurogenetics is dedicated to the diagnosis, treatment and monitoring of individuals and families with (monogenic or genomic) genetic conditions in which the main manifestations are related to developmental delay, or are secondary to degeneration or dysfunction of the central or peripheral nervous systems. One should distinguish clinical neurogenetics from the term *neurogenetics* , which has a broader meaning, and is understood as the science that studies genetic variations that have repercussions on any neurological function. Thus, neurogenetics encompasses clinical neurogenetics and the study of multifactorial or polygenic diseases 1 2 ( Figure 1 ).

The aim of the present narrative review is to propose a guide and an organization of the diagnostic reasoning applicable to clinical neurogenetics. Considering the marked scientific and technological advances that occurred during the last decades in this field, the present article describes a general proposal that can be used as a model for the diagnostic approach to neurogenetic diseases.

## CLASSIFICATION OF NEUROGENETIC DISEASES

Before the presentation of the hypotheses regarding diagnostic reasoning, it is relevant to better understand how neurogenetic diseases are classified. The main clinical features for this classification include topography and age at onset.

### Classification according to topography

**Figure 2:**
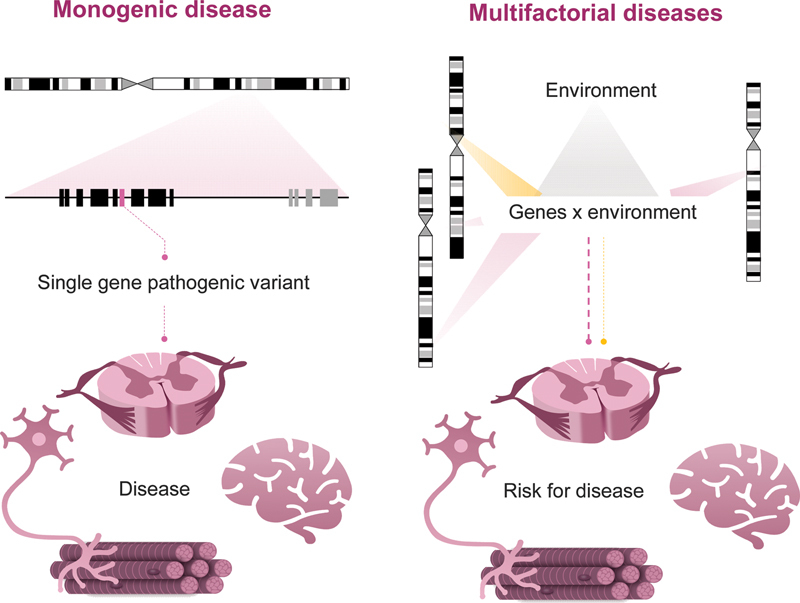
General models of monogenic and multifactorial diseases. The gray shape indicates the effect of the environment on the risk of developing a particular condition. Reddish forms indicate variants in genes that confer risk, and yellow forms indicate variants in genes that confer protection for a given condition. The thickness of the shapes indicates the size of the variant's effect on the development of the phenotype. Abbreviation: CNS, central nervous system.

One of the most practical classifications of neurogenetic diseases follows topographic diagnosis, as showed in Figure 2 .

The biggest challenge regarding this classification for a non-specialist is the anamnesis collection and the correct practice, acquisition, and interpretation of the neurological examination. In many cases, for a correct topographical diagnosis, requesting additional tests, such as nerve conduction studies, electromyography and neuroimaging, is necessary. The proper accomplishment of this process is the initial step, which is fundamental for the adequate classification and development of a diagnostic hypothesis.

### Classification according to the age at the onset of symptoms

Around 90% of monogenic diseases start in childhood, only around 10% start after puberty, and 1%, in adulthood. 3 Although there are fewer late-onset monogenic conditions, they tend to be more prevalent in the general population, due to the autosomal dominant inheritance in many of these conditions, affecting many individuals per family.

In general, neurogenetic diseases with congenital or childhood onset are related to: 1) defects in the development of the nervous system (such as defects in neuronal migration), usually static and non-progressive conditions; 2) metabolic diseases, which may start prematurely with signs of intoxication or involve intermittent conditions associated with episodes of greater metabolic demand, such as energy metabolism defects, or may even have a degenerative course, as seen in defects in the metabolism of complex molecules; 4 and 3) neurotransmitter defects, of varied presentation, with signs of encephalopathy, epilepsy, and pyramidal and extrapyramidal syndromes. 5 Most diseases with a late onset or beginning in adolescence have neurodegeneration as their main mechanism, usually presenting a slowly progressive course.

The classification of neurogenetic diseases according to the age at onset is more useful if considered together with the disease course and within the topographic diagnostic groups. This data on age is essential when evaluating the performance of molecular techniques to obtain a definitive diagnosis. For example, today we know that the performance of next-generation sequencing (NGS) panels or exome sequencing (ES) differs according to the age group for the same syndrome/topography (cases of infantile onset ataxia or cerebellar hypoplasia present better performances with NGS panels or ES when compared with late-onset hereditary ataxias, for example). 6 7 8 9

## ORGANIZATION OF DIAGNOSTIC REASONING IN NEUROGENETICS

The diagnostic reasoning in neurology is usually organized in the following order: the syndromic diagnosis, which consists of the recognition of the signs and symptoms that make up the different neurological syndromes (pyramidal and extrapyramidal motor syndromes, cognitive syndrome, etc.), followed by topographic or anatomical diagnoses (in which an attempt to find a single-site lesion that better explains the patient's signs and symptoms is made), and, finally, the etiological diagnosis, in which the clinical course of presentation and the patient's demographic and epidemiological data provide the main clues to identify the underlying cause (vascular, infectious, neoplastic, degenerative, iatrogenic, congenital, immune, functional etc.). 10 11 In the case of neurogenetic diseases, or in the case of an evaluation requested by the neurogeneticist, the process is slightly different, as its order is reversed. In other words, the process is biased since the etiological diagnosis, with the assumption that a genetic condition was already suspected as the etiological basis for the disease, or that it is necessary to rule out a genetic condition.

Thus, to organize the diagnostic reasoning in neurogenetics, we suggest an approach different from that of classical neurology, in which we will start by answering the following questions:

1) What is the main neurological syndrome? (Examples: pyramidal, lower motor neuron, cerebellar and parkinsonian motor syndromes, etc.).
2) Are there supporting neurological syndromes? If so, which ones? (Examples: pyramidal motor, lower motor neuron, cerebellar motor, and parkinsonian motor syndromes etc.).
3) Is there involvement of other organs and tissues (especially unusual findings)? If so, which ones? (Examples: visceromegaly, ichthyosis, telangiectasia, achalasia, xanthoma, heart disease, cataract, retinopathy etc.).
4) Is the clinical course of the condition compatible with a neurogenetic disease? Remember that most neurogenetic conditions beginning after childhood have a degenerative course, which is slow, progressive, and insidious, and that conditions that alter the development of the nervous system tend to have a static course. Exceptions to this clinical course rule are rare, and many of these conditions have a stereotyped presentation course that may assist in the suspected diagnosis (examples: rapid-onset dystonia-parkinsonism associated with mutations in the *ATP1A3* gene). 12
5) What is the likely pattern of inheritance? (Examples: autosomal dominant, autosomal recessive, X-linked, mitochondrial, and sporadic; Table 1 ).

**Table 1:** Fundamental questions when obtaining family history

**Is there family recurrence?** The recurrence of similar and unusual conditions in the same family is a fundamental clue for the diagnosis of genetic conditions. When encountering recurrence, try to collect information about as many affected family members as possible. Sometimes, it will be necessary to examine these family members to confirm the reliability of the information. Remember that the family history of common illnesses, such headache, hypertension, diabetes mellitus, and late-onset dementia, should be evaluated very cautiously, because in most cases this information will not be relevant enough to modify the diagnostic suspicion.
**Is there consanguinity?** When collecting the family history, watch out for consanguineous marriages in the family of the proband (suggesting an autosomal recessive inheritance), and try to define the degree of kinship.
**Geographic isolates?** The proband's place of birth, and their parents and grandparents may also be of relevance to the diagnosis, both because of the possibility of some form of more frequent condition in that region (such as founder effect of Machado-Joseph disease in Southern Brazil, 30 familial amyloid polyneuropathy in Rio de Janeiro, 31 and families with amyotrophic lateral sclerosis related to the *VAPB* gene in the Southeastern Region of the country 32 33 ), and to reveal potential distant unknown consanguinity.
**Possibility of hidden recurrence?** -Early death of the parent: pay attention to the age of death of the parents. Remember that the early death of one of the parents (before the expected age of manifestation of the disease) can hide the family history of the condition. -Doubtful paternity: doubtful paternity can also hide family history. We must bear in mind the possibility of doubtful paternity and, if there is subsequent confirmation of a genetic condition in which this scenario is possible, the matter must be approached carefully, at the right time and conditions. Remember that dubious parenting is not of medical interest itself, and its search can lead to family conflicts.
All of the aforementioned data will assist in establishing the likely inheritance mechanism. However, we emphasize that the absence of these factors does not exclude the possibility of genetic conditions for several reasons (examples: de novo variants, which are new spontaneous variants emerging from a germline cell of one of the parents or in the fertilized egg; incomplete penetrance, which means that not everyone who has a given genotype will express the phenotype; variable expressivity, which means that subjects with the same genotype may have different disease severity or different clinical pictures; common frequency of carriers of pathogenic variants for some autosomal recessive diseases in the general population, as spinal muscular atrophy; compound heterozygosity for recessive disease etc.). Therefore, clinicians should refrain from saying that the family history is negative, but should rather inform the answers to the questions raised above.

After performing the aforementioned process, we organize the diagnostic reasoning into “positive” findings, that is, those that are present in the affected patient or family, and “negative” findings, that is, relevant findings that are absent in the affected patient or family. It is important to note that the list here does not have to be long. In general, the main positive findings, added to a specific positive finding (if any), are crucial for the development of diagnostic hypotheses, and the negative findings help to rule out several differential diagnoses or markedly reduce their probabilities. Even if the use of new diagnostic computational technologies becomes a reality in the clinical practice, it is unlikely that it will replace the critical role of the clinician in the correct performance of anamnesis, family history, and physical examination and its interpretation in the context of neurogenetics.

## DIAGNOSTIC HYPOTHESES AND REQUEST FOR CONFIRMATORY EXAMS

To develop the diagnostic hypotheses, we start with the organization of the “positive” and “negative” findings, as we have already seen, and then we ask:

1) What is the patient's main neurological syndrome? In other words, does the patient mainly present intellectual disability, cerebellar ataxia, spastic paraparesis, dystonia etc.?
2) Is there a main diagnostic suspicion (the sum of the clinical findings suggests a single or a few etiologies)?
  - Yes: request a diagnostic confirmation test.
  - No: if there are specific findings for this group of conditions that you have not evaluated, consider scheduling a reevaluation of the patient and their affected family members, or requesting a paraclinical exam that can assist in the best phenotypic characterization. If the reassessment and/or clinical characterization is not specific or there are multiple probable causes, consider ordering tests that evaluate multiple genes or regions simultaneously.
  3) Do I have sufficient knowledge of genetics to adequately choose and explain the test I will request, as well as its possible implications, prior to ordering it? Do I have sufficient knowledge to interpret the test report in most scenarios?
  - Yes for both questions: request the exam.
  - No for any of the questions: consider referring the case to another specialist that better meets the aforementioned requirements.
Of note, it is paramount to know the molecular basis of the main diagnostic suspicions before choosing the proper genetic test. Bear in mind that some types of disease-causing variants can be identified only by specific techniques, as in Friedreich ataxia, in which 90% of the cases are homozygous for a GAA trinucleotide expansion in intron 1 of the *FXN* gene. In this case, if we inadvertently choose to perform ES or an NGS panel for ataxias to solve the diagnosis, the requested exam will not be able to confirm the diagnosis nor to exclude it, because the pathogenic variant is located in an intronic region, and the trinucleotide expansions have not yet been correctly identified by the currently-available NGS platforms.
4) What is the cost and what is the average time to obtain the test results? It is essential to discuss openly with the patient and their family about the need for the exam and its possible involved costs.
5) What is the impact of the result for the patient and their family? At the time of the request, it is important to discuss the impact of confirming or excluding a given diagnosis for the family. Remember that if the main diagnostic hypothesis does not have a modifying treatment at the time of the request, it is always possible to wait for the diagnostic confirmation at a more opportune moment and, even if there is no modifying treatment, there is always some form of treatment, which, in general, does not depend on the specific etiology, but on the clinical characterization of the symptoms (which was performed throughout the evaluation).
6) Have the treatable causes for this condition been ruled out? Never forget sporadic and treatable genetic conditions! Even if they are not the most likely hypotheses, they should be the first to be investigated, except in situations in which another diagnostic suspicion is very high.

**Figure 3:**
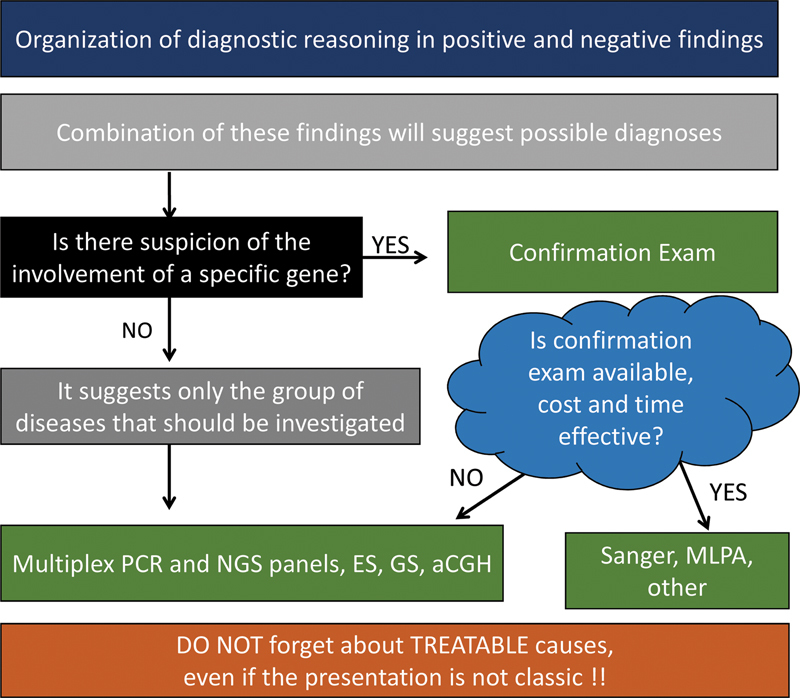
Organizing diagnostic hypotheses and ordering tests in neurogenetics. Abbreviations: aCGH, array comparative genomic hybridization; ES, exome sequencing; GS, genome sequencing; MLPA, multiplex ligation-dependent probe amplification; NGS, next-generation sequencing; PCR, polymerase chain reaction.

In Figure 3 the proposed workflow for the development of a diagnostic hypothesis and to request confirmatory tests is summarized.

## INTERPRETATION OF THE RESULTS OF GENETIC TESTING

After receiving the result of the requested genetic testing, we must interpret the variants related to the monogenic conditions that were found. According to the 2015 guidelines of the American College of Medical Genetics and Genomics (ACMG), 13 the variants should be classified into pathogenic, likely pathogenic, of uncertain significance (VUS), likely benign or benign. The most controversial category, which will generate greater doubts, is that of the VUS. In the case of variants that are probably pathogenic and probably benign, the chance that the classification is correct (pathogenic or benign) is greater than 90%, which is considered strong enough to confirm or refute a diagnosis. There are criteria considered to be very strong, strong, moderate and supportive for this classification, and their combination defines in which category the variant is classified. These criteria will probably be updated in a few years; thus, it is suggested to always check if medical societies have published new guidelines for the classification of variants such as the ACMG or other initiatives.

In general, the starting point for variant analysis is the search for the allele frequency in the population, which must be performed in databases such as that of the Exome Aggregation Consortium (ExAC), which has now been replaced by the Genome Aggregation Database (gnomAD, available at http://gnomad.broadinstitute.org/ ), 14 which provides whole exome sequencing data for ∼ 123,000 unrelated individuals and whole genome sequencing data for ∼ 15,000 unrelated individuals from population studies or with specific multifactorial diseases; the database 1,000 genomes, and the Brazilian Initiative on Precision Medicine (BIPMED, available at http://www.bipmed.org/ ) 15 or the Brazilian Online Mutation Archive (Arquivo Brasileiro Online de Mutações, ABRAOM, in Portuguese, available at http://abraom.ib.usp.br/ ). 16 It is important to note that the ExAC database was made available in October 2014, and the gnomAD, two years later, and it is common that, before 2014, researchers considered the absence of a specific variant in a population of ∼ 100 control subjects as one of the pathogenic criteria. With the availability of these large databases, it became frequent to reclassify variants previously considered to be pathogenic and recorded in databases such as the Human Gene Mutation Database into benign variants.

After knowing the allelic frequency of the variant, it is necessary to evaluate the type of the variant found. The most important data for the classification of the pathogenicity of variants is the presence of a null variant (nonsense, frameshift and canonical splicing site) related to a condition in which the loss of function is the causal pathophysiological mechanism. The canonical splicing site is located 1 to 2 nucleotides before the start or after the end of a given exon. Mutations that may alter the splicing site, but that are not in these positions, receive a prediction score for splicing alteration, in item PP3. The PP3 is a support item for the pathogenicity classification of the variant, that is, it has a weak weight for this determination. It is in this item that the different *in silico* algorithms will be used to predict pathogenicity score. Several algorithms can be used for this purpose, including Sorting Intolerant from Tolerant (SIFT), 17 Polymorphism Phenotyping, version 2 (PolyPhen-2), 18 Combined Annotation-Dependent Depletion (CADD), 19 Mutation Taster, 20 Mendelian Clinically Applicable Pathogenicity (M-CAP), 21 and Rare Exome Variant Ensemble Learner (REVEL) 22 for missense variants; SpliceAI, 23 Human Splice Finder 3.1, 24 and Exonic Splicing Enhancer Finder (ESEfinder) 25 for the prediction of splicing sites; and Genomic Evolutionary Rate Profiling, version 2 (GERP ++), 26 for the prediction of nucleotide conservation. The article by Richards et al., 13 contains a fuller list of the algorithms that can be used. However, bear in mind that overestimating the result of the prediction of pathogenicity by the *in silico* analysis can lead to misdiagnosis.

Nevertheless, if the variant is considered a VUS, there is some information that can help change this classification to the side of pathogenicity or benignity. One of them, which is usually not feasible in the neurogenetics clinical practice (except for some muscular dystrophies), is the so-called functional analysis. For this analysis, it is necessary to establish, in an in vitro or in vivo model, whether or not the variant found causes damage to the gene or to the gene product involved. Although functional analysis is the criterium that generates evidence of greater strength of pathogenicity or benignity of the variant, the two most feasible strategies in the clinical practice are: 1) the search in specific databases of variants of the condition or gene in question, which may be more informative than generic databases (examples Leiden Muscular Dystrophy pages versus Human Gene Mutation Database for variants in muscular dystrophies), as well as contact with international specialists in the condition (including the variant deposition in GeneMatcher, available at https://genematcher.org/ ), who may reveal information from other cases with the same variant not yet reported in the literature; and 2) segregation analysis, which will look for the variant found in other affected family members and in family members who have no symptoms. Depending on the number of available and genotyped individuals in a family, segregation analysis may be classified either as supporting or strong criteria of pathogenicity. 13 27 Segregation analysis can also provide strong evidence of benignity when the variant is absent in a family member with the same clinical syndrome. 13 Whenever feasible, one should try to assess as many individuals as possible with the same pedigree. There are similar classification systems for microdeletions and microduplications. 28

## REVIEWING THE DIAGNOSTIC HYPOTHESIS

Even if the entire process has been properly performed, a considerable number of patients will not have their final diagnosis determined. Thus, if, after the investigation, the diagnostic tests are negative, consider redoing the diagnostic process from the beginning, as some cognitive biases 11 might have occurred:

1) Ordering of information: the order in which information is presented influences our decision-making process, so when sorting a list of problems or diagnostic hypotheses, we tend to place more value on the information and hypotheses listed first. The excessive weight given to this order can lead to errors in the diagnostic process.
2) Heuristic anchoring: in this case, the main hypothesis is considered so strong that the clinician cannot elaborate alternative hypotheses or refuses changes in the main hypothesis with the discovery of new clinical facts.
3) Impact of recent diagnoses: the recent diagnosis of any condition will increase the chance of considering this diagnosis for other cases closely evaluated. It is common to hear colleagues saying that a rare disease usually comes in pairs. Be careful, because this statement is fallacious, and it only implies that when we detect a rare condition in a given patient, we will pay more attention to that diagnosis in the period that comes soon after. This can be positive and lead to a correct diagnosis, or it can induce distortions in the development of different diagnostic hypotheses and the weights given to them, sometimes leading to the request of unnecessary tests, prolonging the patient's diagnostic struggle.
4) Representative heuristics: it occurs when we lose the perspective of the frequency of the condition in the population, and we use other factors to guide our hypotheses. It is quite possible that we make this mistake frequently in clinical neurogenetics, because in our assessment we generally assume that it is likely to be a neurological genetic condition, and these conditions are much rarer in the population than common multifactorial diseases. Anyway, this type of bias should be relativized or better contextualized, because in the aforementioned situation, the population scenario would be the prevalence of the diagnosis of a neurogenic condition among the referrals made to this specialty. Certainly, the prevalence of neurogenic conditions in this context will be markedly higher than the prevalence in the general population.
5) Blind obedience: it happens when we overestimate the authority of third parties (example: one is not allowed to change the main diagnostic hypothesis, if it was developed by a more experienced professional) or the result of diagnostic tests. In the latter case, we can mention as examples the attitude of organizing the diagnostic hypotheses based on the results of nerve conduction studies, even if these are in conflict with the topographic diagnosis of the neurological examination; and blindly relying on the confirmation of the diagnosis by molecular analysis, even when the evolution of the condition over time contradicts the diagnosis made. We have already seen some cases of errors in molecular diagnosis, either in the collection of the exam, or by errors inherent to the technology used or to the interpretation of its results. These errors were only discovered with clinical observation over time and because there was a strong clinical impression that the patient's condition could not be explained by the findings of the genetic testing.

We should also bear in mind that genetic testing is relatively recent, and that genomic techniques, which are revolutionizing the diagnosis and the discovery of new conditions, are even more recent. So, it is possible that the condition of the patient is indeed monogenic, but that its genetic base has not yet been described.

### The role of clinical follow-up in cases without a definitive diagnosis

Certainly, at the end of the investigation of a patient with a suspected neurogenetic condition, we will have defined the main syndromic diagnosis and we will be able to establish which group of conditions fits the patient's presentation, even without a definitive molecular diagnosis. In other words, we will also have a probable clinical diagnosis, with a degree of certainty similar to that of multifactorial diseases. The search for treatable conditions as the initial steps of the investigation will not only help to avoid delays in the institution of effective treatment, but will also help to remove the urge to establish the final diagnosis. Thus, we need to make it clear to the patient that the specific diagnosis will not often change their treatment and that the symptomatic treatment measures that are already being applied are independent from the etiological definition.

However, we emphasize that most of the time we will try to reach the definitive diagnosis. In general, confirming the etiological diagnosis (regardless of whether there is specific treatment) provides some comfort to the patient or family member, since the anguish of thinking a wrong path is being followed or that time is being wasted before the start of a treatment would be mitigated by the definitive diagnosis. In addition, with the definitive diagnosis, we will have more accurate information to carry out the proper genetic counseling, and to provide data on prognosis, education and rehabilitation, as well as the possibility of inclusion into clinical trials. 29

In conclusion, the present article was designed to guide, facilitate and organize diagnostic reasoning in neurogenetics, resulting in a more rational development of hypotheses and in greater resoluteness and efficacy in the diagnostic investigation. In addition to experiencing a revolution in the diagnostic aspects of genetic conditions in recent years with the advent of the NGS and array comparative genomic hybridization (aCGH), another revolution, in the treatments that modify the clinical course of these conditions, in some cases including the description of mutation-specific treatments, is also emerging. In this scenario, it is very likely that in the coming years even greater importance will be placed on the confirmation of the molecular diagnosis of neurogenetic diseases for the establishment of the therapeutic plan. Assisted reproduction techniques such as preimplantation diagnosis, which depends on the previous molecular diagnosis of the family condition, and which may be one of the options for couples at risk of having children affected by the same condition, are also expected to perform even better, with lower costs, and with greater access, especially in public health care systems of different countries.
